# Thermodynamic Consistency of the Cushman Method of Computing the Configurational Entropy of a Landscape Lattice

**DOI:** 10.3390/e23111420

**Published:** 2021-10-28

**Authors:** Samuel A. Cushman

**Affiliations:** USDA Forest Service, Rocky Mountain Research Station, Flagstaff, AZ 86004, USA; Samuel.cushman@usda.gov

**Keywords:** entropy, Boltzmann, configuration, landscape, Cushman method

## Abstract

There has been a recent surge of interest in theory and methods for calculating the entropy of landscape patterns, but relatively little is known about the thermodynamic consistency of these approaches. I posit that for any of these methods to be fully thermodynamically consistent, they must meet three conditions. First, the computed entropies must lie along the theoretical distribution of entropies as a function of total edge length, which Cushman showed was a parabolic function following from the fact that there is a normal distribution of permuted edge lengths, the entropy is the logarithm of the number of microstates in a macrostate, and the logarithm of a normal distribution is a parabolic function. Second, the entropy must increase over time through the period of the random mixing simulation, following the expectation that entropy increases in a closed system. Third, at full mixing, the entropy will fluctuate randomly around the maximum theoretical value, associated with a perfectly random arrangement of the lattice. I evaluated these criteria in a test condition involving a binary, two-class landscape using the Cushman method of directly applying the Boltzmann relation (s = klogW) to permuted landscape configurations and measuring the distribution of total edge length. The results show that the Cushman method directly applying the classical Boltzmann relation is fully consistent with these criteria and therefore fully thermodynamically consistent. I suggest that this method, which is a direct application of the classical and iconic formulation of Boltzmann, has advantages given its direct interpretability, theoretical elegance, and thermodynamic consistency.

## 1. Introduction

Recently there has been a rapid increase in research to develop theory and methods for calculating the entropy of landscape patterns (e.g., [1,2,3,4]). The original applications of entropy in ecological research as well as image processing were based on Shannon entropy [5]. These methods are limited in that they are not explicitly sensitive to different configurations of the system (e.g., they measure compositional rather than configurational entropy, sensu [1]). Application of entropy measures to landscape ecology requires explicit attention to and quantification of spatial patterns (configuration in addition to composition). Recently, there has been a concentrated effort to move away from non-spatial information-entropy approaches, rooted in Shannon entropy, to explicit calculation of Boltzmann entropy of landscape patterns, which is rooted in counting the frequency of microstates across a full distribution of possible landscape configurations [1,2,6]. The important distinction between these two lines of research is that the latter directly focuses on the entropy of different configurations of landscape patterns.

Several approaches have been proposed to directly quantify the configurational entropy of landscapes, including an approach to directly apply the Boltzmann relation to permuted landscape patterns (the Cushman method [1,2]) and a number of more complex approaches, such as using multi-resolution analysis (the Gao method [3,4,7]) and also other alternative entropy formulations (such as Wassenstein entropy [8,9,10]).

All of these approaches have theoretical strengths and differ in complexity and the measurements they produce. Until now there has been little information, however, on the thermodynamic consistency of the different methods. The Gao method(s) have been evaluated and found to be partly thermodynamically consistent, following modifications [6]. Additionally, the Wassenstein approach has been evaluated and found to be consistent with several criteria of thermodynamic consistency following clarification and modification by [10]. In this paper, I evaluate the thermodynamic consistency of the Cushman method of calculating the configurational entropy of a landscape mosaic.

The Cushman method [1,2] is a direct application of the iconic Bolzmann relation (s = klogW) to measuring landscape entropy. Namely, entropy is proportional to the logarithm of the number of microstates producing a given microstate. The microstates used in the Cushman method are unique arrangements of a landscape lattice, defined as a raster mosaic of different classes. The macrostate in the Cushman method is the total edge length between pixels of different cover class. Cushman [1] proposed this direct application of the Boltzmann relation using these definitions of microstate and microstate. Cushman [2] also demonstrated that the probability distribution of edge length across all microstates was Gaussian and that the entropy function was parabolic, with maximum entropy corresponding to spatial randomness and minimum entropy corresponding to maximum aggregation or maximum dispersion.

For the Cushman [1,2] method of computing the configurational entropy of a landscape lattice to be thermodynamically consistent, I propose it must meet three criteria. First, the computed entropies must lie along the theoretical distribution of entropies as a function of total edge length, which Cushman [2] showed was a parabolic function following from the fact that there is a normal distribution of permuted edge lengths, that the entropy is the logarithm of the number of microstates in a macrostate, and that the logarithm of a normal distribution is a parabolic function. Second, the entropy must increase over time through the period of the random mixing simulation, following the expectation that entropy increases in a closed system. Third, at full mixing, the entropy will fluctuate randomly around the maximum theoretical value, associated with spatially random arrangement of the lattice.

## 2. Methods

I evaluated he thermodynamic consistency of the Cushman [1,2] method for two scenarios, the first of which consisted of starting from a perfectly aggregated landscape lattice (two homogeneous patches) and the second of which consisted of starting from a perfectly dispersed landscape lattice (checkerboard), with 50% cover in each of two classes in each scenario. These represent two extremes of low entropy, as [2] definitively showed that entropy is lowest when landscape patterns are far from random mixing and that both highly aggregated and highly dispersed patterns are far from the expectation produced by random mixing and therefore low in entropy.

The first step of the analysis was to confirm that the distribution of total edge lengths for a two-class lattice was normally distributed and that the entropy curve for such a lattice was parabolic. I evaluated the fit of a range of two-class landscapes with 50% cover of each class to the expectations of the normal probability density function and the parabolic density curve. Specifically, I evaluated landscapes of dimensionality 10 × 10, 20 × 20, 40 × 40, 80 × 80, 128 × 128, and 160 × 160. The purpose of this was to demonstrate, following [2], that all dimensionality of a two-class landscape with 50% coverage followed the expectation. I evaluated this using the methods developed by [1,2]. Specifically, [2] showed that the frequency of microstates (arrangements of the landscape lattice) that produce the same macrostate (total edge length in a landscape lattice) were normally distributed, with a peak centered at the edge length expected under random mixing. Further, [2] showed that the entropy function of this microstate distribution was parabolic, following the Boltzmann relation, which posits that entropy is directly proportional to the logarithm of the number of microstates that produce the observed microstate, and that given that the logarithm of a normal distribution is a parabola, the entropy function is parabolic.

Second, I conducted a random mixing simulation following the approach of [4], in which, at each time step, 1% of the pixels were randomly switched. This is the simplest version of a random mixing experiment and represents unconstrained random mixing, which is an appropriate null model to evaluate thermodynamic consistency of methods to calculate landscape entropy. I calculated the curve of expected distribution of configurational entropy for a lattice of 128 × 128 dimensionality and 50% coverage in each of two classes and plotted the observed configurational entropy of the landscape lattices produced by the two simulation experiments (maximally aggregated and maximally dispersed starting condition). At each time step of the simulation, I calculated the total edge length and configurational entropy of the resulting lattice. The entropy was calculated following the Cushman [2] method of calculating the mean and standard deviation of total edge lengths out of 1,000,000 permutations of the 128 × 128 lattice and using this normal probability density function to compute the number of microsates in each macrostate (possible length of total edge). This enabled the calculation of the configurational entropy of each observed total edge length in the mixing experiment. Specifically, it enabled direct evaluation of the second two criteria mentioned above: that the mixing experiment will produce change toward maximum entropy, as measured by the method, and that, at equilibrium, the system will fluctuate indefinitely around the state of maximum entropy.

## 3. Results

### 3.1. The Distribution of Total Edge Length for a Two-Class Lattice of 128 Rows and Columns and 50% Cover of Each of Two Classes Is Normally Distributed and the Entropy Curve Is Parabolic

**Figure 1:**
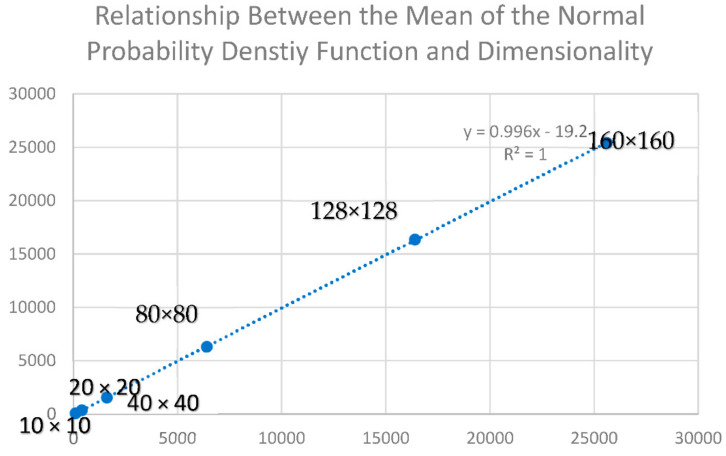
Plot of the relationship between the mean value of the normal probability density function of permuted total edge lengths for landscape lattices with two classes of equal proportionality and having a range of dimensionality (10 × 10, 20 × 20, 40 × 40, 80 × 80, 128 × 128, and 160 × 160 cells). The simulation experiment was performed on landscapes with 128 × 128 dimensionality. The *y*-axis shows mean of the normal probability density function, and the *x*-axis shows the number of cells in the landscape (nrows*ncols).

Consistent with [2], there was a perfect linear relationship between the number of cells in the landscape and the mean of the normal probability density function of permuted total edge length of the landscape lattice (Figure 1). Confirming the results of [2], this analysis demonstrates that this linear relationship has a slope of 1 and explains 100% of the variance in the mean of the normal probability density function of total edge length. Thus, there is consistency in the relationship between the mean of the microstate distribution and the dimensionality of the landscape lattice, satisfying the first criterion.

**Figure 2:**
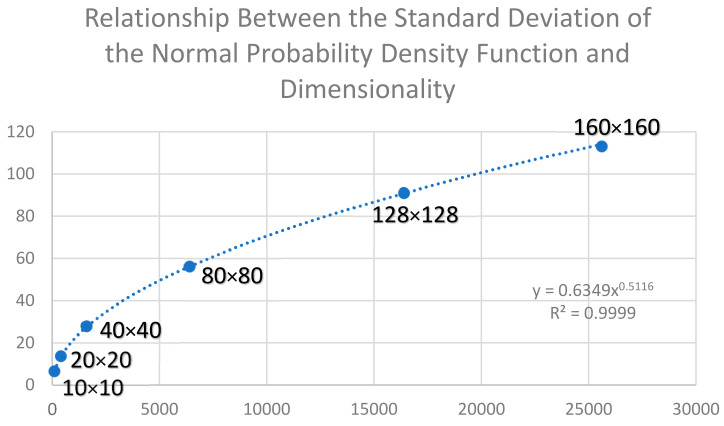
Plot of the relationship between the standard deviation of the normal probability density function of permuted total edge lengths for landscape lattices with two classes of equal proportionality and having a range of dimensionality (10 × 10, 20 × 20, 40 × 40, 80 × 80, 128 × 128, and 160 × 160 cells). The simulation experiment was performed on landscapes with 128 × 128 dimensionality. The *y*-axis shows standard deviation of the normal probability density function, and the *x*-axis shows the number of cells in the landscape (nrows*ncols).

Also consistent with the findings of [2], the standard deviation of the normal probability density function of permuted total edge lengths was a power function of the dimensionality of the landscape, with the standard deviation being directly related to the square root of the number of cells in the landscape (nrows*ncols; Figure 2). This power function had an exponent of 0.5, indicating a perfect parabolic relationship, and explained 100% of the variance in the distribution of calculated entropies across the distribution of permuted microstates.

### 3.2. The Distribution of Total Edge Lengths Produced by the Mixing Experiments Follows the Expected Distribution of the Cushman Method of Computing Configurational Entropy

**Figure 3:**
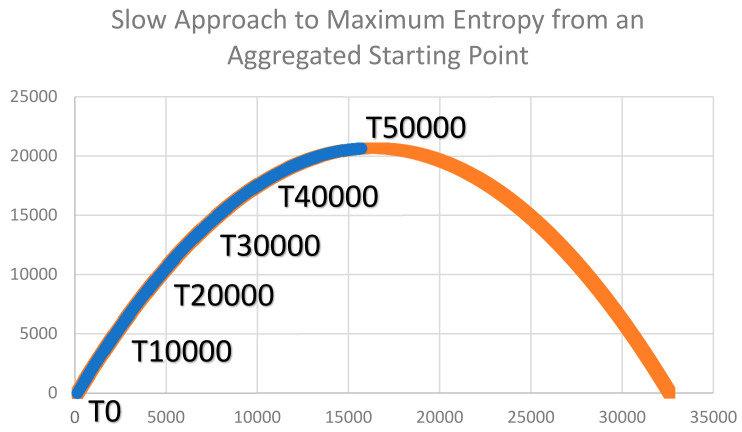
Plot of the theoretical distribution of entropy of a 128 × 128 cell lattice with 50% cover of each of two cover classes (orange line) and the distribution of observed entropy across the mixing experiment from an aggregated starting condition (blue line). The *y*-axis is entropy of the lattice. The *x*-axis is total edge length of the lattice. The time-steps of the simulation experiment are labeled on the graph (T0—starting condtion; T10000—10,000th time-step; T20000—20,000th time step; T30000—30,000th time step; T40000—40,000th time step, T50000—50,000th time step).

**Figure 4:**
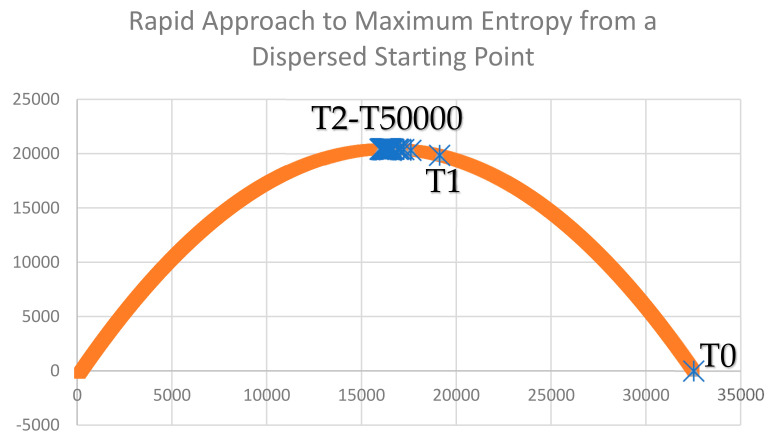
Plot of the theoretical distribution of entropy of a 128 × 128 cell lattice with 50% cover of each of two cover classes (orange line) and the distribution of observed entropy across the mixing experiment from a fully dispersed starting condition (blue markers). The *y*-axis is entropy of the lattice. The *x*-axis is total edge length of the lattice. The time-steps of the simulation experiment are labeled on the graph (T0—starting condtion; T1—1st time-step; T2-T50000—2nd to 50,000th time step).

I calculated the curve for the expected distribution of configurational entropy for a lattice of 128 × 128 dimensionality and 50% coverage in each of two classes and plotted the observed configurational entropy of the landscape lattices produced by the two simulation experiments (aggregated and dispersed starting points). The first criteria for thermodynamic consistency of the Cushman [1,2] method of computing the configurational entropy of a landscape lattice is that the mixing experiment would produce configurational entropy predictions that follow the theoretical distribution. This was the case for both the aggregated and dispersed scenarios (Figure 3 and Figure 4). Specifically, the observed entropies of the lattices produced by the mixing experiment perfectly followed the expected theoretical distribution (parabolic function of total edge length).

### 3.3. The Entropy of the Landscape Lattice Increases through the Mixing Experiment

The second criteria for evaluating the thermodynamic consistency of the Cushman [1,2] (2016, 2018) method of calculating the configurational entropy of a landscape lattice is that the entropy must increase through the course of the mixing experiment in the simulated closed system. This was the case for both scenarios of the mixing experiment (aggregated and dispersed starting condition). Specifically, under the aggregated starting condition, there was a continuous increase in observed entropy, with the initial condition of a perfectly aggregated two-homogeneous patches having entropy very near zero (since there are only four possible configurations with that low amount of total edge; Figure 3). The entropy increased steadily, reaching a value very near the theoretical maximum at the end of the simulation experiment (50,000 time-steps). In the case of the mixing scenario beginning in a perfectly dispersed (checkerboard) condition, the entropy of the initial condition was very near zero, following [1,2], which observed that there are exceptionally few arrangements of a lattice that are perfectly dispersed. The entropy rapidly increased toward the maximum possible entropy in this scenario across the simulation time (Figure 4). Specifically, in a single time step, the entropy increased to over 90% of the theoretical maximum, and from the second to the 50,000th time step, the entropy fluctuated near the theoretical maximum.

### 3.4. The Entropy Fluctuates around the Theoretical Maximum Following Complete Mixing

**Figure 5:**
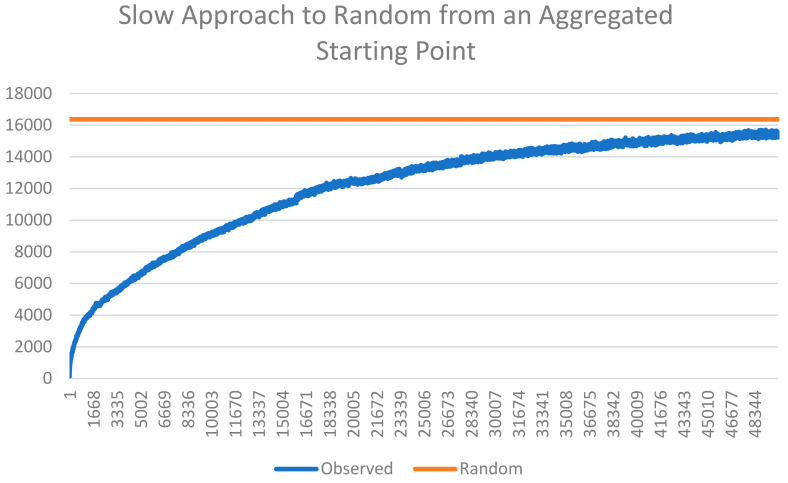
Plot of the approach of observed total edge length of the lattices produced by the mixing experiment from an aggregated starting condition to a fully randomized state. The *y*-axis is the total edge length. The *x*-axis is the time step of the mixing simulation. The blue line is the observed total edge length. The orange line is the total edge length of the expected value of the distribution of 100,000 permutations of a lattice of 128 × 128 cells and 50% area of two cover classes.

**Figure 6:**
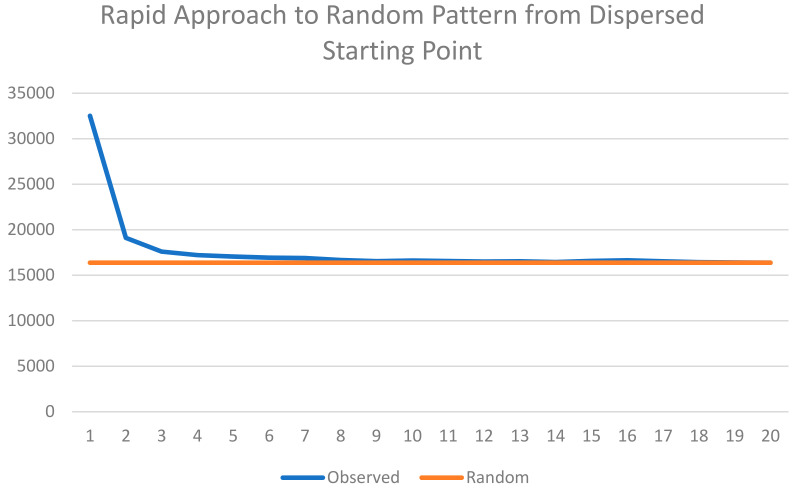
Plot of rate of approach of observed total edge length of the lattices produced by the mixing experiment from perfectly dispersed starting condition to a fully randomized state. The *y*-axis is the total edge length. The *x*-axis is the time step of the mixing simulation. The blue line is the observed total edge length. The orange line is the total edge length of the expected value of the distribution of 100,000 permutations of a lattice of 128 × 128 cells and 50% area of two cover classes.

The third criteria for evaluating the thermodynamic consistency of the Cushman [1,2] method is that when a lattice is completely mixed (fully randomized), it should have a value near the maximum theoretical value of the entropy distribution and should fluctuate indefinitely around this maximum theoretical value with further random mixing. We observed this to be the case in the scenario of the dispersed starting condition but not the aggregated starting condition. This is because of the very large difference in the time needed to achieve full mixing in the two scenarios (Figure 5 and Figure 6). Specifically, in the aggregated starting condition scenario, the mixing experiment resulted in a slow, continuous approach to a fully randomized condition but never fully achieved full randomization within the 50,000 time steps (Figure 5). In contrast, the dispersed scenario very rapidly approached randomness within four time steps (Figure 6) and subsequently fluctuated randomly very near the value of maximum theoretical entropy, as required for thermodynamic consistency.

## 4. Discussion

The recent interest in the theory and methods of calculating configurational entropy of landscape patters is a recognition of the fact that landscape ecology is the science of pattern and scale and their influence on ecological processes, and that thermodynamics, the second law in particular, are the fundamental drivers of all natural change and therefore all ecological processes. Thus, connecting entropy and the second law to ecological theory is critically important to give the science of ecology a true theoretical basis and improve its predictive ability based on first principles of energetics and physics [11].

Landscapes have patterns that have been quantitatively measured for many years, such as through landscape metrics [12,13], of which there are hundreds available that measure a plethora of different attributes of landscape structure. Some landscape ecologists question the utility or the need of developing new measures of landscape patterns based on entropy. However, existing landscape pattern methods are generally unrelated (at least formally) to the second law of thermodynamics, and therefore formalizing a general and consistent approach for measuring the entropy of landscape patterns is essential to provide a link between thermodynamics and landscape pattern–process relationships.

Among the methods developed to calculate the configurational entropy of landscapes, those based on Boltzmann entropy have the most promise and are theoretically the most appealing. Specifically, there has long been application of Shannon information entropy [5] in image analysis, informatics, community ecology, and other processes [14], but existing methods of calculating information entropy are not formally sensitive to different configurations of a landscape (they measure the entropy of different degrees of composition but are not sensitive to different configurations given a particular composition). The Bolzmann relation (s = klogW) is the classic measure of entropy of systems and has direct interpretability. This is the reason that Cushman [1,2] proposed direct application of the Boltzmann relation to calculating landscape entropy through random mixing experiments to calculate the distribution of the frequency of microstates across a range of macrostates of landscape pattern and suggested total edge length in a landscape lattice mosaic as a measure of the microstate of landscape pattern most relevant for consideration.

A number of other, more complex, formulations of landscape entropy have been proposed. For example, [3,4,5,6,7] proposed several modifications of multi-scale aggregation methods to calculate entropy. Recently, Wassenstein entropy has been proposed as an attractive approach [8,9,10]. These formulations of landscape entropy may not be fully thermodynamically consistent in that they generally do not recognize that entropy is highest in the zone of random mixing, have not shown that the distribution of microstates is Gaussian, and do not show that the entropy function is parabolic with low entropy in both maximally dispersed and maximally aggregated conditions.

There are a number of methods that have been proposed to evaluate thermodynamic consistency in chemical and physical sciences. For example, [15] describes tests to evaluate the consistency of chemical data related to the excess molal properties of binary mixtures. That method applies the Gibbs–Duhem equation to derive the expected range of mole fraction conditions at thermodynamic equilibrium, which is useful to provide quality control evaluation to identify experimental or measurement errors producing chemical measurements that are not thermodynamically plausible. The evaluation presented in this paper is slightly different, in that it is not a method to evaluate empirical measurements relative to known thermodynamic chemical processes. Rather, this evaluation is intended to theoretically evaluate a particular method for calculating spatial entropy itself. Thus, it differs in two important ways. First, the goal is to confirm theoretical thermodynamic consistency of the entropy measure itself rather than in empirical data. Second, given this goal, the method appeals to first principles of the second law, namely that entropy must increase in the closed system under stochastic change. Additionally, the method assesses consistency in terms of the distribution of microstates and the shape of the entropy function and whether the random mixing experiment produces patterns of change that are consistent with the expectations for these. The approach and criteria used in this paper are highly similar to those applied in [6], namely that the random mixing experiment will increase entropy from any starting condition. I add the additional two criteria mentioned above to further clarify consistency relative to the expectations of the distribution of microstates and the shape of the entropy function, which are fundamental assumptions of the Cushman method to directly apply the Boltzmann relation for quantifying the spatial entropy of landscape mosaics.

The Cushman method [1,2] is a direct application of the classical Boltzmann formulation of entropy, which gives it theoretical attractiveness as being as close as possible to the root theory and original formulation of entropy. It is also attractive for its direct interpretability and ease of application. This paper extends [1,2] by showing that the configurational entropy of a landscape mosaic is fully thermodynamically consistent based on all three criteria I tested. Namely, this analysis confirms that the distribution of microstate frequency (as measured by total edge length in a landscape lattice) is normally distributed; it confirms that the entropy function from this distribution of microstates is parabolic; it confirms a linear relationship between mean value of the normal distribution of microstates and the dimensionality of the landscape mosaic; it confirms the power function relationship (parabolic) between the dimensionality of the landscape and the standard deviation of the normal distribution of microstates. These latter two findings are reported here for the first time and provide additional theoretical guidance for practical application of the Cushman method across landscapes of different extent and dimensionality. Cushman [2] previously showed how to generalize the method to landscapes of any size and number of classes, and the new findings reported here provide guidance into how the parameters of the microstate distribution and entropy function change systematically with landscape extent.

Additionally, this paper shows that the Cushman method directly applying the Bolzmann relation is fully consistent with expectations under a random mixing experiment. Specifically, I showed in this analysis that, starting from low entropy states of different configuration (maximally aggregated and maximally dispersed), a random mixing experiment resulted in approach toward maximum entropy, as calculated by the Cushman method. Interestingly, I found a large difference in the rate at which maximum entropy is approached in the random mixing experiment for the two different low entropy patterns in the initial condition. For aggregated initial patterns, there was a slow, gradual approach to maximum entropy (at the condition of full spatial randomness). In contrast, from the starting condition of maximum dispersed landscape patterns, the mixing experiment very rapidly approached maximum entropy. The distribution of the entropy function for both initial conditions shows that both are equally low in entropy at the beginning, but they differ fundamentally in the time-function for how rapidly entropy equilibrates to its maximal state. This suggests that aggregated patterns have higher thermodynamic inertia than dispersed patterns, while they both have equally low entropy. This has important implications for ecological theory and applications of entropy research, given that the high thermodynamic inertia of aggregated patterns suggests that aggregated landscape structures are more resistant to change as a result of management, disturbance, or other perturbation, in terms of the entropy of the landscape.

This analysis focused on a two-class categorical landscape mosaic as a case study. This was intentional to be consistent with the historical focus of landscape ecology on categorical patch mosaics [16,17] and their patterns [18]. It was also performed to be consistent with and to build on the past work developing the Cushman method of direct application of the Boltzmann relation for calculating landscape entropy [1,2]. However, recent work (e.g., [19]) has shown that the Cushman method applying the Boltzmann relation applies equally well to calculating the configurational entropy of surfaces and point patterns. Cushman [19] also showed that applying the method to surfaces and point patterns is thermodynamically consistent in terms of producing normally distributed microstates and parabolic entropy functions. That work did not conduct a time-process mixing experiment, but the consistency of the application to gradients and point patterns in the distribution of microstates and the shape of the entropy function strongly suggests that the method is fully consistent for all landscape patterns, including landscape conceptual models (sensu [18]) based on landscape mosaics, gradients, and point patterns.

Future work should formally explore the linkage between formal analysis of entropy of living and physical systems with additional topics in complexity theory, such as logical depth [20,21]. Logical depth is a measure of complexity of a system based on the length or complexity of computer code required to simulate its numerical attributes and the processing time required to run this code. Cushman [14] suggested that there should be a relationship between entropy and complexity, such as that measured by logical depth. Specifically, he suggested that a system with maximum entropy would have the lowest level of complexity based on logical depth, given maximum entropy equates to maximum randomness and computer code to simulate randomness is simple and quick to run. Maximum entropy, conversely, corresponding to high aggregation or systematic dispersion, would require more complex simulation processes and therefore represent a system of greater logical depth. It would be valuable to explore the generalization of the relationships between logical depth and configurational entropy for point patterns, gradients, and landscape mosaics.

## Data Availability

Data are available directly from the author.
